# Supplementary material for: Human class B1 GPCR modulation by plasma membrane lipids
Source: Commun Biol. 2026 Jan 8;9:317. doi: 10.1038/s42003-025-09445-2 (PMC12936092; doi:10.1038/s42003-025-09445-2)
Supplement: Supplementary file 2 — Description of Additional Supplementary Files [file 42003_2025_9445_MOESM2_ESM.docx]

Description of Additional Supplementary Files

File name: Supplementary Movie 1

Description: **Movie showing the top interaction sites of cholesterol and PIP2 identified from PyLipID.** 3D alignment of top ranked cholesterol and PIP_2_ interaction sites across all 15 receptors from cgMD simulation. Sites are aligned to the active (green) and inactive (blue) states of the CTR.

Spheres represent i) PIP_2_ headgroups for the top ranked interaction sites extracted from simulations of active (dark magenta) and inactive (light pink) states, ii) cholesterol headgroups for the top ranked interaction sites extracted from simulations of active (dark orange) and inactive (light orange) states.
